# Electrosprayed Multi-Core Alginate Microcapsules as Novel Self-Healing Containers

**DOI:** 10.1038/srep34674

**Published:** 2016-10-03

**Authors:** Iee Lee Hia, Pooria Pasbakhsh, Eng-Seng Chan, Siang-Piao Chai

**Affiliations:** 1 Advanced Engineering Platform, Mechanical Engineering Discipline, School of Engineering, Monash University Malaysia, 47500, Bandar Sunway, Selangor, Malaysia; 2 Advanced Engineering Platform, Chemical Engineering Discipline, School of Engineering, Monash University Malaysia, 47500, Bandar Sunway, Selangor, Malaysia

## Abstract

Alginate microcapsules containing epoxy resin were developed through electrospraying method and embedded into epoxy matrix to produce a capsule-based self-healing composite system. These formaldehyde free alginate/epoxy microcapsules were characterized via light microscope, field emission scanning electron microscope, fourier transform infrared spectroscopy and thermogravimetric analysis. Results showed that epoxy resin was successfully encapsulated within alginate matrix to form porous (multi-core) microcapsules with pore size ranged from 5–100 μm. The microcapsules had an average size of 320 ± 20 μm with decomposition temperature at 220 °C. The loading capacity of these capsules was estimated to be 79%. Under *in situ* healing test, impact specimens showed healing efficiency as high as 86% and the ability to heal up to 3 times due to the multi-core capsule structure and the high impact energy test that triggered the released of epoxy especially in the second and third healings. TDCB specimens showed one-time healing only with the highest healing efficiency of 76%. The single healing event was attributed by the constant crack propagation rate of TDCB fracture test. For the first time, a cost effective, environmentally benign and sustainable capsule-based self-healing system with multiple healing capabilities and high healing performance was developed.

For the past decade, self-healing epoxy composites have attracted immense attraction, particularly for longevity and possibly cost reduction^1^. Different self-healing approaches have been reported including extrinsic and intrinsic self-healing. Extrinsic self-healing requires moderate temperature whereas external stimuli is needed for the latter^2^,^3^. Extrinsic self-healing can be disintegrated into capsule and vascular-based self-healing systems^2^,^4^,^5^,^6^. Both systems function in a similar approach where restoration of damages is done by the prefilled healing agents within either capsules or vascular-based containers.

Different microvascular networks have been introduced including hollow glass fibers^7^, hollow tube reinforced shape memory polymer^8^, conductive microwire/glass microtubes network^9^, fugitive wax scaffold^10^ and polyacrylonitrile (PAN) nanofibres^11^. These continuous vascular networks were interconnected which contributed to the multiple healing ability. Although vascular-based healing systems could provide healing for larger damaged area, demonstrating multiple healing ability and the vascules act as a form of structural reinforcement, their fabrication process is rather sophisticated and the micro-range vascular networks limit the healing to microcracks^1^,^7^,^12^,^13^,^14^.

Likewise, most of the capsule-based self-healing systems showed unfavorable one-time healing and utilized toxic formaldehyde for capsules fabrication^15^,^16^,^17^,^18^,^19^,^20^. Studies have shown that formaldehyde may cause cancer and adverse health effects, thus it has been regulated in the work place for years^20^. Furthermore, synthesis of these formaldehyde containing capsules such as urea-formaldehyd^2^, polyurethane/urea-formaldehyde^21^ and poly(melamine-formaldehyde)^16^ capsules through *in situ* polymerization process is complicated because temperature and pH alteration are required. Other capsules such as conducting polymer^22^ and polystyrene nanocapsules^23^ were reported, but they were used specifically for corrosion inhibitor. Furthermore, these self-healing microcapsules do not possess the properties as a reinforcement to the whole composite system. Thus, efforts on exploiting for greener, sustainable and more efficient capsules are substantial as well as improving the healing frequency provided by the self-healing system.

Herein, we proposed a novel formaldehyde free capsule-based self-healing system with alginate biopolymer as the carrier for epoxy resin to form porous (multi-core) microcapsules. Due to the amphoteric and active properties of amine hardener^24^, it is difficult to find a suitable hardener that does not react with alginate. Thus, this study will focus on studying the suitability and possibility of using alginate as the encapsulating material for epoxy monomer. Besides, the self-healing ability of the synthesized capsules were evaluated through *in situ* healing of impact and tapered double cantilever beam (TDCB) specimens. To date, we are the first using electrospraying method to fabricate microcapsules for self-healing composites as well as using a biopolymer as the self-healing capsule materials. The alginate biopolymer we proposed is a natural biopolymer extracted from brown seaweed and its well-known properties such as biocompatibility, biodegradability and non-toxicity make it a great encapsulation medium^25^. Generally, alginate hydrogels are formed by ionic crosslinking with Ca^2+^ or Ba^2+^ ions^26^,^27^ which is a simple and cost effective process without temperature and pH alteration.

## Materials and Methods

### Materials

Two different types of epoxy resin (diglycidyl ether of bisphenol A) were used, EPIKOTE 828 (12 000–14 000 cps at 25 °C) for epoxy matrix and ARALDITE 506 (500–700 cps at 25 °C) for capsule core materials. Both epoxy resin and calcium chloride (analytical grade) were purchased from Sigma-Aldrich. The epoxy matrix hardener, diethylenetriamine, DETA was purchased from BASF. Sodium alginate (Manugel GHB, FMC Biopolymer, UK) with medium range molecular mass of 37% β-D-mannuronic acid (M) and 63% α-L-guluronic acid residues (G) was used in this study due to higher elastic modulus with higher G residues^26^.

### Microcapsules Synthesis

**Figure 1:**
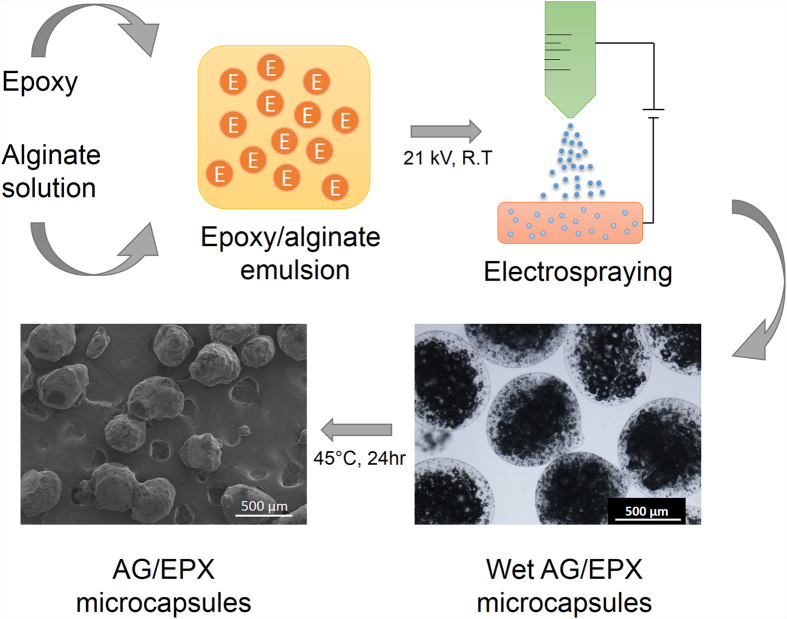
Schematic illustration of multi-core AG/EPX microcapsules synthesis process.

Figure 1 illustrates the synthesis process of alginate/epoxy AG/EPX multi-core microcapsules which was carried out at ambient temperature. Initially, alginate solution (2 w/v %) was prepared by mixing alginate and distilled water using a mechanical stirrer for 30 min at 500 rpm. The solution was then sealed and left overnight for degassing. Calcium chloride solution (2 w/v %) was prepared by dissolving calcium chloride in distilled water. Subsequently, epoxy resin was gradually added to the mechanically stirred alginate solution (2 w/v %) to form an (10 v/v %) oil-in-water emulsion at 500 rpm for 30 min. The AG/EPX microcapsules were then produced through electrospraying method as developed by Nedović *et al*. in 2001^28^. During electrospraying, epoxy/alginate emulsion was pumped (NE-1010, New Era) through a G24 stainless steel needle with a flow rate of 40 mL/hr. An electrostatic potential of 13–21 kV was connected to the needle whereas the CaCl_2_ gelling solution was grounded. The distance between the needle tip to the calcium chloride solution was kept at 7 cm. AG/EPX wet microcapsules were formed once the epoxy/alginate emulsion was crosslinked with calcium chloride solution. Subsequently, the AG/EPX wet microcapsules were rinsed 3 times with distilled water, sieved and dried at 45 °C for 24 hr to form AG/EPX multi-core microcapsules. Same procedures were applied to produce blank calcium alginate microcapsules.

### Characterization of Microcapsules

AG/EPX wet microcapsules were analyzed using an Upright Metallurgical Microscope (Olympus BX41M, LED) at various magnification scale and the size was measured using Image J software. The average diameter was calculated from 30 measurements. FTIR (Nicolet iS10) was used to determine the chemical structure of the microcapsules. The FTIR spectra for each sample was carried out in the range of 500–4000 cm^−1^. For each spectrum, 64 scans were collected at a resolution of 4 cm^−1^. Thermogravimetric analysis (TGA Q50) was used to determine the thermal stability of the prepared microcapsules by heating the samples from 25 °C to 600 °C at a rate of 10 °C/min under nitrogen atmosphere. The loading capacity (LC) of epoxy within AG/EPX microcapsules was estimated based on Eq. (1):


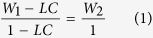


where W_1_ is the total weight loss of AG/EPX microcapsules and W_2_ is the weight loss of blank calcium alginate microcapsules calculated from the TGA results^29^. Surface morphology and cross section of AG/EPX microcapsules were examined using field emission scanning electron microscope (SU8010 FE-SEM, Hitachi) after sputter coating the samples with platinum. For the cross section study, the ruptured microcapsules were rinsed with acetone to remove the encapsulated epoxy resin.

### Specimen preparation

**Figure 2:**
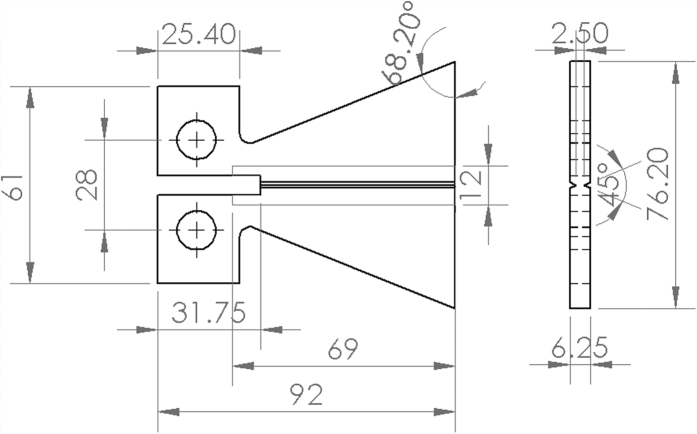
A schematic drawing of localized TDCB geometry and dimensions. All the dimensions are in the unit of millimetre.

Two types of specimens, Charpy impact (100 mm × 10 mm × 4 mm) and localized TDCB (Groove length = 60.25 mm) as shown in Fig. 2 were fabricated to assess the healing of the composites. Neat epoxy specimens were fabricated by mixing EPIKOTE 828 with DETA at a ratio of 100:12 whereas epoxy composites were fabricated by mixing the neat epoxy with different loading of AG/EPX microcapsules (10 wt%, 15 wt%, 20 wt% and 30 wt%) uniformly prior degassing and then poured into silicon rubber mold to cure for 24 hr at 30 °C. For TDCB specimens, the degassed epoxy was poured into silicon rubber mold with rubber inserts in the middle section (Fig. 2) and let it cured overnight. The rubber inserts were then removed and filled with mixture of epoxy and AG/EPX microcapsules at different loading (15 wt%, 20 wt% and 30 wt%) followed by curing for 24 hr at 30 °C. Each batch of samples consists of five specimens to provide the mean values. Two types of control specimens were fabricated, neat epoxy without microcapsules and epoxy infiltrated with 20 wt% of alginate microcapsules.

### Assessment of Healing through Impact Test

After curing, the specimens were removed from the mold and lightly polished in order to have smooth surface. A V-shaped notch of 2 mm was made at the midpoint of the impact specimens with a notch-tip radius of 0.25 mm^30^. The specimens were then undergone Notched Charpy Impact test (CEAST 9050) compliance to ISO 179 with pendulum energy of 5 J and a span of 60.0 mm. The instrumented impact strength was determined and used for healing efficiency evaluation. The broken two pieces of epoxy composites were then undergone *in situ* healing test by applying 0.1 μL of DETA on one of the broken surface and aligned the broken pieces carefully on the healing platform for healing to take place. Minor pressure was applied on the specimens and there were undergone healing at 40 °C for 48 hr. After healing, the respective instrumented impact strength was tested again. Neat epoxy and epoxy with 20 wt% blank alginate microcapsules as the control samples were undergone the same healing process as stated above. The healing efficiency of instrumented impact strength recovery is calculated according to the relative impact energies which is introduced by Hayes *et al*.^31^,


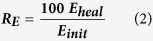


where E_heal_ and E_init_ represent the post-healing and initial instrumented impact strength whereas R_E_ is the percentage recovery in impact strength.

### Assessment of Healing through TDCB Fracture Test

For the TDCB fracture test, a pre-crack was formed by tapping a fresh razor blade into the front part of molded groove of the TDCB specimens before testing. Subsequently, the specimens were pin-loaded on the Instron universal testing machine (Instron 5982) under displacement rate of 1 mm/min at ambient temperature until the crack was fully propagated through the groove. The peak load was then determined for healing efficiency evaluation. Similarly, *in situ* healing was performed on the specimens by applying 0.2 μL of DETA on one of the broken pieces before joining the broken pieces together to heal at 40 °C for 48 hr. Same procedures were undergone to test and heal the control specimens. The evaluation of healing efficiency, η is defined as the ratio of fracture toughness of healed specimen, K_IC_^healed^ to the virgin specimen, K_IC_^virgin^, which is then reduced to the ratio of peak loads at fracture^32^.


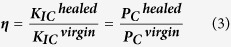


### Statistical analysis

Analysis of variance (ANOVA) was performed using commercial software to evaluate the significant differences between the means of impact strength and peak load with respect to the AG/EPX microcapsules loading. In every cases, *P* < 0.05 was considered as statistically significant.

## Results and Discussion

**Figure 3:**
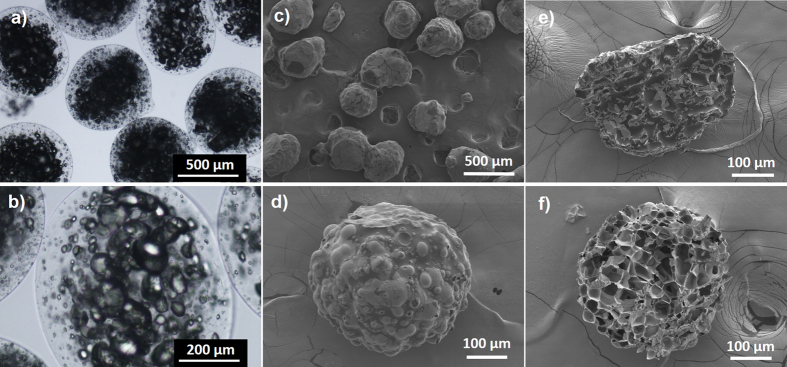
(**a**,**b**) Microscopic images of wet AG/EPX hydrogels showing multi-core epoxy is being encapsulated inside calcium alginate matrix. (**c**,**d**) FESEM images of dry AG/EPX microcapsules with bumpy and rough surface as well as the (**e**) respective cross section showing epoxy resin is being encapsulated (darker regions) within the (**f**) porous internal structure after washing with acetone. The multi epoxy cores are separated by the porous calcium alginate matrix.

As shown from Fig. 3a,b, multi-core AG/EPX wet microcapsules were formed with multiple epoxy resin cores being encapsulated within calcium alginate matrix. The mean diameter was determined to be 575 ± 25 μm. As water within the wet capsules evaporated, the capsule size shrank about 29% from the original volume (Fig. 3c,d) and the diameter was determined to be 320 ± 20 μm. Field-emission scanning electron microscope (FESEM) micrographs show that these dried microcapsules have irregular shapes and bumpy surfaces as water from the surrounded calcium alginate matrix evaporated and shrank during the drying process, whereas the multi-core epoxy resin did not, thus leaving the capsules to have bumpy surface which was the shapes of the encapsulated multi-core epoxy resin. During the cross section study, porous internal structure was observed on the AG/EPX microcapsule after removing the encapsulated epoxy resin by acetone (Fig. 3e,f) and the pore size was found to be in the range of 5–100 μm through FESEM. To date, most of the reported studies on self-healing microcapsules are core-shell type where a layer of shell is formed around a single core ingredient^21^,^33^,^34^. Generally, this feature leads to single healing event as once the capsules are ruptured, most of the releasing healing agents (mixture of epoxy and hardener) are polymerized and thus leaving insufficient healing agents for subsequent damage at the same spot^35^.

**Figure 4:**
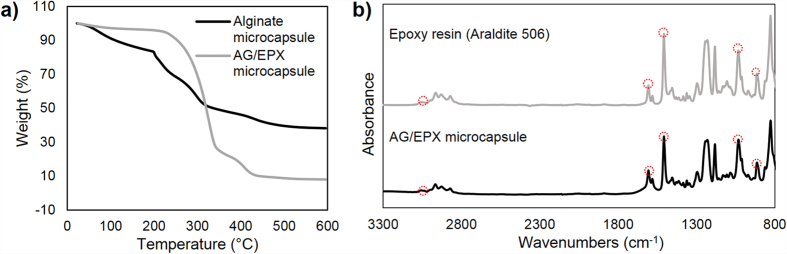
(**a**) Thermogravimetric analysis (TGA) mass loss curves of blank alginate and AG/EPX microcapsules. (**b**) FTIR spectra of epoxy resin (Araldite 506) and AG/EPX microcapsules.

TGA was carried out to investigate the thermal stability of blank calcium alginate and AG/EPX microcapsules as shown in Fig. 4a. It can be seen that the thermal decomposition for both type of capsules are different from each other. For the calcium alginate microcapsules, the first mass loss of 17% from 50–200 °C corresponded to the adsorbed moisture and degradation of glycosidic bonds. The subsequent 40% of mass loss from 220–430 °C corresponded to the further decomposition of glycosidic bond. As the temperature increased, the generated residues which contained carbonaceous char and calcium carbonate acted as the protective barrier and prevented the blank alginate microcapsules from further decomposition^36^. For AG/EPX microcapsules, the first 5% of mass loss from 25–210 °C represents the adsorbed moisture and degradation of glycosidic bonds. The capsules further loss about 70% of its mass from 210–350 °C. Such a drastic increase in its mass loss was due to the decomposition of uncured epoxy resin at 300 °C^14^. The further mass loss was corresponding to the residues of carbonaceous char and calcium carbonate. The total mass loss for AG/EPX microcapsules was estimated to be 91.9% whereas for calcium alginate microcapsules was 61.7% and the LC of the monomer microcapsules was calculated to be 78.9%. Subsequently, FTIR spectroscopy analysis was carried out on epoxy resin and dried AG/EPX microcapsules (Fig. 4b) shows peaks at 3057 cm^−1^, 1607 cm^−1^, 1508 cm^−1^, 1033 cm^−1^ and 915 cm^−1^ where the corresponding chemical groups (Table 1) are confirmed to be diglycidylether of bisphenol A^37^. This confirms that epoxy was successfully encapsulated, and no interaction was detected between calcium alginate matrix and epoxy resin as no new peak was formed.

**Figure 5:**
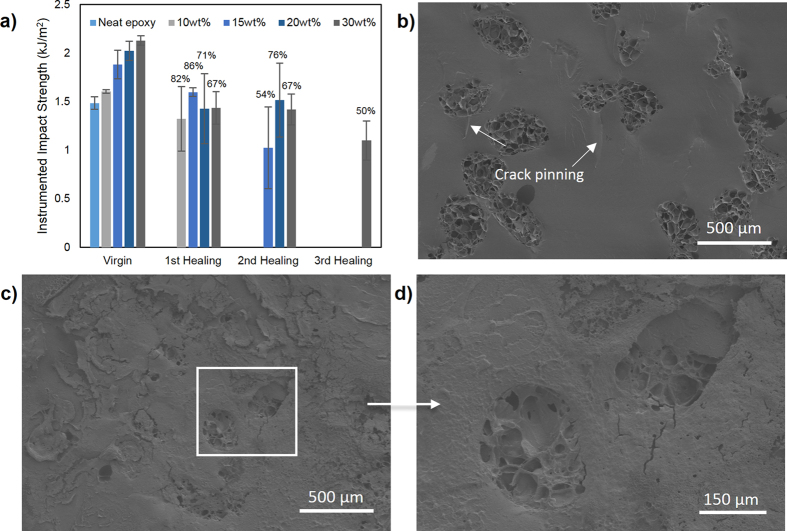
(**a**) Instrumented impact strength recovery of epoxy composites at different AG/epoxy microcapsules loading. The respective healing efficiencies are stated above the graphs. FESEM images of the fracture surface of a (**b**) impact specimen before healing followed by (**c**) healed impact specimen and (**d**) its respective enlargement image. All the specimens were acetone washed and contained 20 wt% of AG/EPX microcapsules.

**Figure 6:**
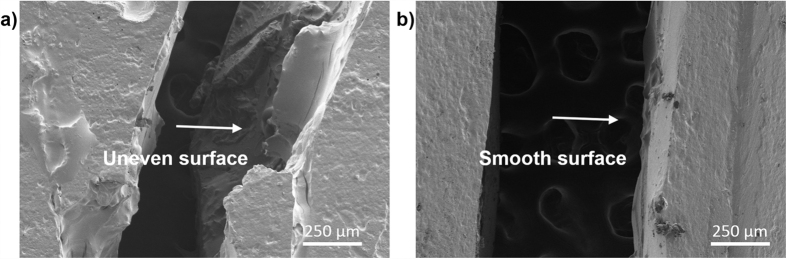
FESEM images (top view) of the fractured (**a**) healed impact specimen and (**b**) healed TDCB specimen, both loaded with 20 wt% of AG/EPX microcapsules. The healed impact specimen showed uneven fractured surface with signs of crack deviation whereas the TDCB specimen showed a smooth fractured surface.

In this study, instrumented impact and TDCB fracture tests were carried out to study the healing ability of these microcapsules. *In situ* healing tests were carried out on both impact and TDCB specimens by manually adding the hardener on the fracture surface. Figure 5a shows the fracture recovery of impact specimens at different capsules loading. As shown, the virgin impact strength of composites increases gradually with capsules loading and the differences between the mean values are significant according to ANOVA at (*P* < 0.05). This is due to the toughening mechanism such as crack pinning occurs through the incorporation of microcapsules as shown in Figure 5b^32^,^38^. These AG/EPX microcapsules acted as reinforcers as they increased the impact absorbed energy during the crack propagation, leading to a higher impact strength. FESEM images in Fig. 5b–d show the fracture surfaces of impact specimens before and after healing. After washing the fracture surface with acetone, the porous internal structure of the microcapsules were revealed as shown in Fig. 5b. Besides, these microcapsules were well adhered to the epoxy matrix, demonstrating the existence of strong adhesive bonding between calcium alginate and epoxy matrix. On the other hand, the impact specimens show repetitive healing with a maximum of three healing cycles and the healing efficiency were more than 50% for every capsules loading (Fig. 5a). It is believed that the porous multi-core structure and the high impact energy exerted on the specimen during the impact test contribute to the multiple healing events. Figure 5c,d show that a layer of polymerized epoxy was formed on top of the fracture plane of a healed specimen. The flaky polymerized epoxy layer partially covered the ruptured microcapsules, revealing the respective porous structures. This indicates that the subsequent impact further breaking the capsules in depth and thus releasing epoxy resin stored underneath. Furthermore, due to the high impact energy and a good adhesion between the calcium alginate and the polymerized epoxy, the subsequent crack propagated within the capsules deviates from the original fracture path as shown in Fig. 6a. This indicates that the subsequent impact test further ruptured the capsules, triggered the releasing of reserve epoxy resin and thus leading to multiple healing. Likewise, both the control specimens showed no sign of healing.

**Figure 7:**
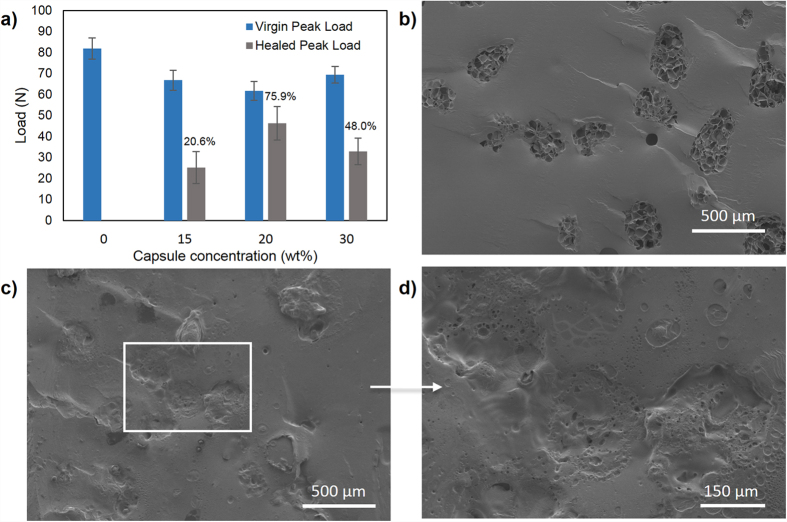
(**a**) Peak load recovery of TDCB specimens at different AG/epoxy microcapsules loading. The respective healing efficiencies are stated above the graphs. FESEM images of the fracture surface of a (**b**) TDCB specimen before healing followed by (**c**) healed TDCB specimen and (**d**) its respective enlargement image. All the specimens were acetone washed and contained 20 wt% of AG/EPX microcapsules.

In another healing assessment, TDCB fracture test were carried out under similar healing conditions by manually applying 0.2 μL of DETA on the fracture surface. Preliminary tests in this study show that no significant healing were observed for capsule loading below 15 wt%. Figure 7a shows the typical load-displacement graph of an *in situ* healed TDCB specimen with AG/EPX microcapsules loading from 15 to 30 wt%. A maximum healing efficiency of 75.9% was achieved at 20 wt% capsule loading, whereas at 15 wt% and 30 wt% capsule concentrations, the peak load recovery was found to be 20.6% and 48.0%, respectively. All the TDCB epoxy composites specimens had slightly lower virgin peak load than neat epoxy specimens but the values were still falling within the range of peak load as compared with other studies^16^,^39^,^40^. Besides, ANOVA showed that the mean virgin peak load difference is insignificant at (*P* < 0.05). Thus, addition of AG/EPX microcapsules has negligible effect on the fracture toughness of the composites. Under FESEM, the fracture surface of TDCB specimen (Fig. 7b) shows that the AG/EPX microcapsules were completely ruptured and the respective porous internal structure was shown after washing with acetone. Although the microcapsules were ruptured completely and epoxy was leaking out as similar for impact specimens, different trend was observed on the healed TDCB fracture surface (Fig. 7c,d). The fracture surface showed cohesion failure where the polymerized epoxy film formed was thicker and smoother. It covered the fracture plane completely including the surface of the fractured capsules which blocked the reserved epoxy underneath. During the subsequent test, the low tensile energy of TDCB fracture test resulted the crack propagates through the original crack path that was created in the first fracture (Fig. 6b). This resulted the crack propagated through the polymerized epoxy on top of the microcapsules, thus preventing the reserved epoxy underneath from flowing out to heal subsequent healing. This explains the single healing cycle attributed by TDCB specimens which is due to the nature of TDCB fracture test whereby the displacement rate is slow and constant. Similarly, all the control specimens showed no signs of healing.

## Conclusion

Epoxy resin has been successfully encapsulated within alginate to form multi-core epoxy microcapsules through a simple yet cost effective electrospraying process. These AG/EPX microcapsules inherited with self-healing ability exhibit good adhesion with epoxy matrix. Remarkably, Charpy impact tests showed multiple healing events up to 3 cycles due to the control release attribute of multi-core capsules system and the high impact energy test. This shows that the epoxy composite system is having a great potential to heal materials even under/after catastrophic failure. In contrast, TDCB specimens showed only single healing cycle which can be explained by the slow and steady crack propagation of the test. It does not trigger the releasing of reserved epoxy from the multi-core microcapsules as there are being blocked by the polymerized epoxy. Future studies will need to focus on embedding both monomer and hardener microcapsules to evaluate the self-healing of a green dual-capsule self-healing system. For the first time, a green biopolymer and an environmental benign, formaldehyde free capsule-based self-healing system which shows multiple healing capability with high healing performance was developed.

## Additional Information

**How to cite this article**: Hia, I. L. *et al*. Electrosprayed Multi-Core Alginate Microcapsules as Novel Self-Healing Containers. *Sci. Rep.* **6**, 34674; doi: 10.1038/srep34674 (2016).

## Figures and Tables

**Table 1:** Characteristics bands of alginate and epoxy microcapsules.

Band (cm^−1^)	Assignment
3057	Stretching of C-H of the oxirane ring
1607	Stretching C = C of aromatic rings
1508	Stretching C-C of aromatic
1033	Stretching C-O-C of oxirane ethers
915	Stretching of C-O of oxirane group
